# Blocking the 5′ splice site of exon 4 by a morpholino oligomer triggers APOL1 protein isoform switch

**DOI:** 10.1038/s41598-018-27104-x

**Published:** 2018-06-07

**Authors:** Amber M. Cheatham, Shamara E. Davis, Atanu K. Khatua, Waldemar Popik

**Affiliations:** 1 Meharry Medical College, Center for AIDS Health Disparities Research, Department of Microbiology and Immunology, 1005 D. B. Todd Blvd, Nashville, TN 37028 USA; 2 Department of Internal Medicine, 1005 D. B. Todd Blvd, Nashville, TN 37028 USA

## Abstract

APOL1 risk alleles G1 or G2 are associated with a kidney disease phenotype exclusively in people of recent African ancestry. Here we show that exon 4 encoding a part of the APOL1 signal peptide is constitutively spliced in major APOL1 transcripts expressed in kidney glomerular and tubular cells. We demonstrate that constitutive splicing of exon 4 results from a suboptimal hnRNP A1 binding motif found in exon 4. Accordingly, a robust binding of hnRNP A1 protein to a consensus hnRNP A1 cis-acting element in exon 4 results in almost complete exclusion of exon 4 from the APOL1 minigene transcripts. Blocking the 5′ splice site at the exon 4/intron boundary with a specific antisense morpholino oligonucleotide excludes exon 4 from the splicing pattern of endogenous APOL1 transcripts. These transcripts are fully functional and produce APOL1 protein isoform that is not normally detectable in podocytes. Together with our previous data showing no cytotoxicity of overexpressed APOL1 isoform lacking exon 4, we propose that morpholino-induced APOL1 isoform switch may provide a new tool to identify *in vivo* molecular mechanism(s) by which risk alleles promote or mediate the kidney disease phenotype.

## Introduction

Increased risk of nondiabetic kidney disease in African Americans has been associated with apolipoprotein L1 (APOL1) gene coding variants G1 and G2, found exclusively in people of recent African ancestry^1,2^. APOL1 variants G1 and G2 confer protection against *Trypanosoma brucei rhodesiense* and possibly other parasites, resulting in high frequencies in Sub Saharan populations^3^. The absence of the APOL1 gene in most primates except humans, gorillas, and baboons has been a major obstacle for testing causality between risk alleles G1 or G2 and development of kidney disease. Recently, however, a transgenic mouse model with a podocyte-specific inducible expression of APOL1 demonstrated that G1 or G2 alleles, but not the wild-type G0 allele, produced albuminuria and glomerulosclerosis, hallmarks of kidney disease^4^. This study confirmed that development of the kidney pathogenic phenotype depends on the expression levels of risk alleles, supporting previous findings that individuals with two risk alleles are at increased risk for kidney disease, as compared to carriers of only one^1,2,5^.

The mechanism by which APOL1 risk alleles mediate cell injury seems to be complex and several mechanisms have been proposed including apoptosis, necrosis, pyroptosis, or autophagic cell death^4,6–11^. Cytotoxicity of APOL1 risk alleles was also shown to result from depletion of cellular potassium and activation of stress-activated protein kinases^12^, interference with endosomal vesicle trafficking^13^, or dysfunctional mitochondria^14,15^. Nevertheless, overexpression of a wild-type G0 variant of APOL1, which has not been associated with an increased risk of the kidney disease, displayed significant cytotoxicity not considerably different from that of G1 or G2 variants^6^. This suggests that APOL1 variants may be inherently toxic but only variant G0 is effectively attenuated *in vivo*. Alternatively, toxicity of risk variants can be potentiated by interaction with additional factors or second hits^16^. One such factor, a soluble urokinase plasminogen activator receptor (suPAR) associated with chronic kidney disease (CKD)^17^, was recently shown to synergize preferentially with G1 or G2 variants for activation of αvβ3 integrin signaling pathway, triggering podocyte injury^18^. Preventing formation of this cell membrane multiprotein complex by lowering expression of extracellular suPAR or inhibiting secretion of intracellular APOL1 could block αvβ3-mediated signaling and reduce the toxicity of APOL1 kidney risk variants.

As reported by several databases including NCBI and Ensembl, the APOL1 gene is composed of seven exons that could potentially produce several alternatively spliced transcripts by selective inclusion of exons 2 and 4^18^. As an exon 4-encoded sequence is part of the APOL1 signal peptide, deletion of exon 4 may likely affect APOL1 secretion and alter its intracellular localization and function. Consequently, although the G1 and G2 mutations are in terminal exon 7, deletion of exon 4 may potentially affect biological activity of APOL1 risk variants. In this context, we have previously demonstrated that cytotoxicity of the APOL1 variant G0 was mitigated by the deletion of the exon 4-encoded sequence^18^.

A process of alternative splicing is regulated by the interaction between cis-acting RNA elements and trans-acting splicing factors. Cis-acting elements include exonic splicing enhancers (ESEs) and intronic splicing enhancers (ISE) that recruit positive trans-acting factors, such as serine/arginine-rich (SR) proteins, which generally promote exon inclusion^19–21^. Exonic splicing silencers (ESSs) and intronic splicing silencers (ISS) recruit negative acting factors, such as heterogeneous nuclear ribonucleoproteins (hnRNPs)^19^. The cooperative or antagonistic interactions between splicing factors affect assembly of the spliceosome, a multi-subunit ribonucleoprotein complex that together with a large number of auxiliary proteins removes introns from pre-mRNA transcripts^22,23^.

Using a splicing reporter minigene, we identified several potential ESEs and ESSs in exon 4 that, due to their suboptimal binding motifs, did not considerably affect exon 4 splicing, suggesting that exon 4 is constitutively spliced. This conclusion was further supported by RNA pull-down assays showing only a minimal binding of SRSF1 and hnRNP A1 to their cognate binding sites in exon 4. We also demonstrate that blocking the 5′ splice sites (5′ss) of exon 4 by an antisense morpholino oligonucleotide results in exclusion of exon 4 from endogenous APOL1 transcripts. In line with our previous observation indicating that exon 4 confers toxicity on APOL1 proteins^24^, we propose that morpholino-induced expression of alternative APOL1 protein isoform lacking exon 4 may provide a new tool to investigate molecular mechanism(s) by which risk alleles promote or mediate the kidney disease phenotype.

## Results

### Splicing pattern of the APOL1 transcripts and expression of APOL1 proteins in human kidney cells

**Figure 1:**
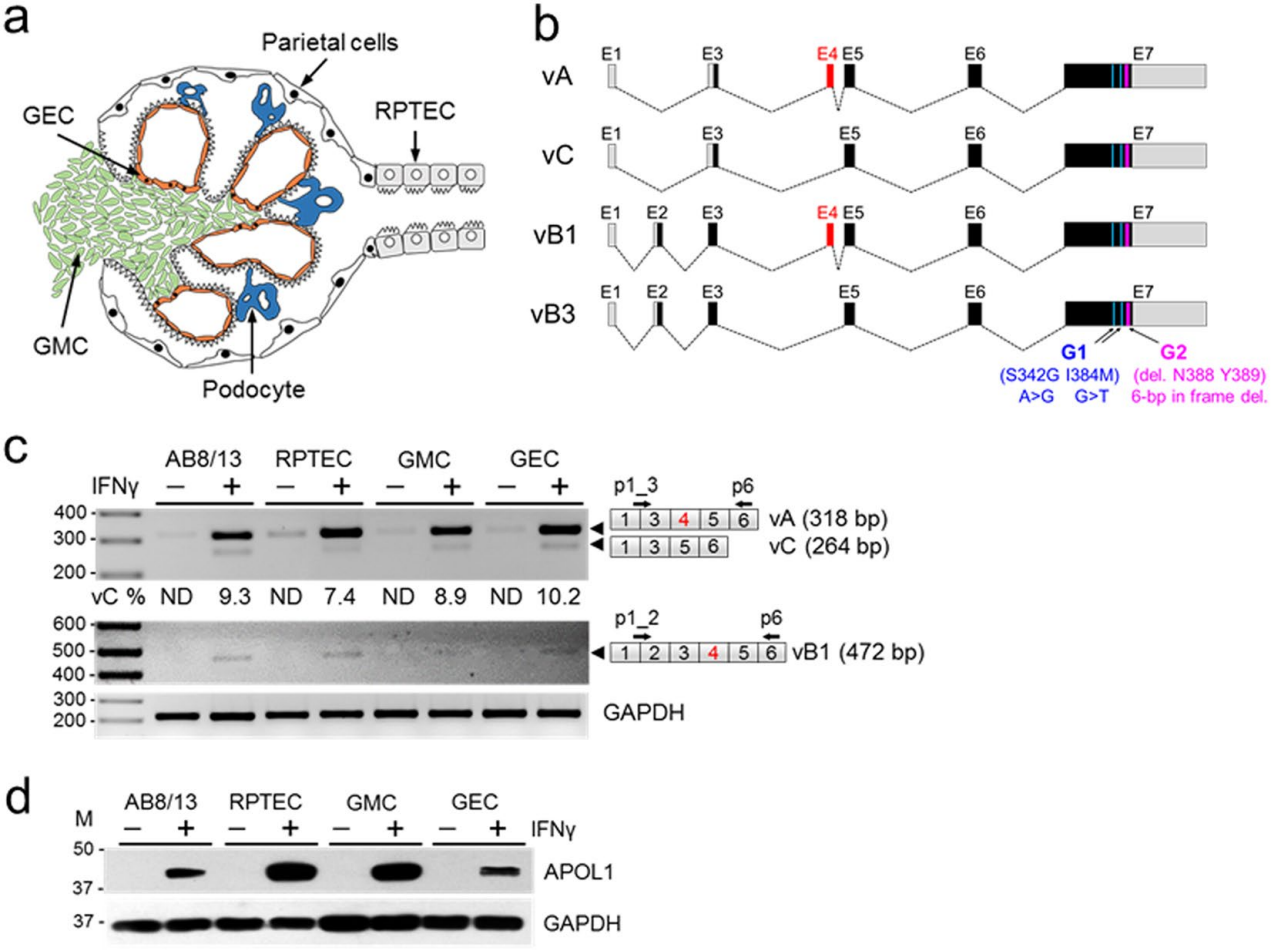
Expression of APOL1 splice variant transcripts and APOL1 proteins in human glomerular and tubular cells. (**a**) Schematic of a glomerulus with indicated four types of glomerular cells: mesangial (GMC), visceral (podocytes), parietal and endothelial cells (GEC), and extraglomerular proximal tubular epithelial cells (RPTEC). (**b**) Schematic representation of major alternatively spliced transcript variants of the APOL1. Gray boxes represent untranslated exons (or parts of exons) and black boxes indicate translated exons. Alternatively spliced exon 4 (red box) is present in variant A and in variant B1. Risk alleles G1 (S342G and I384M) and G2 (deletion N388 and Y386) are present in exon 7. The size of exons and introns are approximate. (**c**) Total RNA was isolated from immortalized human podocytes AB8/13 or primary RPTEC, GMC and GEC treated with (+) or without (−) IFNγ (50 ng/ml) for 24 h and analyzed by RT-PCR using primers specific for APOL1 splice variants A and C (vA, vC; p1_3 and p6) or vB1 or vB3 (p1_2 and p6). APOL1 vB3 was undetectable. Sizes of expected PCR products (bp) of splice variants are indicated (black arrowheads). Percentage of transcripts without exon 4 (vC %) was calculated from densitometric scanning of the vA and vC bands (vC % = [vC/vA + vC] × 100). ND, not determined. Amplified fragments of GAPDH transcripts served as internal control. DNA markers (M) are shown on the left. Representative data from three independent experiments is shown. (**d**) Whole cell lysates from cells treated as in (**c**) were analyzed by western blotting for the expression of APOL1 and GAPDH proteins. Protein size markers (M, kDa) are indicated on the left. Experiments were repeated twice with similar results. The gel images (**c**,**d**) were cropped. The full gel images are shown in Supplementary Fig. 1.

The APOL1 pre-mRNA is composed of seven exons that can be alternatively spliced to generate several APOL1 transcript variants^24–26^. We analyzed the splicing pattern of APOL1 transcripts expressed by human glomerular podocytes AB8/13, primary glomerular mesangial cells (GMC), glomerular endothelial cells (GEC), and renal proximal tubular epithelial cells (RPTECs) (Fig. 1a). The APOL1 splicing pattern was analyzed by RT-PCR using primer sets designed to amplify several APOL1 splice variants (Fig. 1b).

To identify exon 2-negative APOL1 transcripts, which include exon 4 (variant A, NM_003661) or lack exon 4 (variant C, NM_001136541), we used a forward primer spanning a boundary region between exon 1 and exon 3 (p1_3), and a common reverse primer complementary to a nucleotide sequence in exon 6 (p6). Exon 2-containing APOL1 variants, with exon 4 (vB1, NM_145343) or without exon 4 (vB3), were amplified using a forward primer spanning a boundary between exon 1 and 2 (p1_2), and p6 (Fig. 1c).

Unstimulated kidney cells express low levels of APOL1 transcripts and proteins^24^, therefore cells were stimulated for 24 h with IFNγ to increase expression of APOL1. RT-PCR amplification with a primer set p1_3/p6 produced two DNA products (Fig. 1c). The predominant product migrated on a gel according to the size predicted for exon 4-positive APOL1 variant A (vA, 318 bp) and a minor product corresponded to exon 4-lacking variant C (vC, 264 bp). Exon 4-positive splice variant B1 (vB1, 472 bp) represented <5% of total APOL1 transcripts and exon 4-lacking splice variant B3 was hardly detectable under these conditions. Previously, we confirmed identity of the PCR amplicons corresponding to vA and vC by DNA sequencing^24^. Consistent with RT-PCR data, immunoblot analysis demonstrates that stimulation with IFNγ significantly increased the otherwise very low levels of APOL1 proteins in unstimulated kidney cells (Fig. 1d). In summary, these data indicate that human kidney glomerular and tubular cells predominantly express APOL1 splice transcripts with exon 4 (vA).

### Generation and validation of an APOL1 splicing reporter minigene

**Figure 2:**
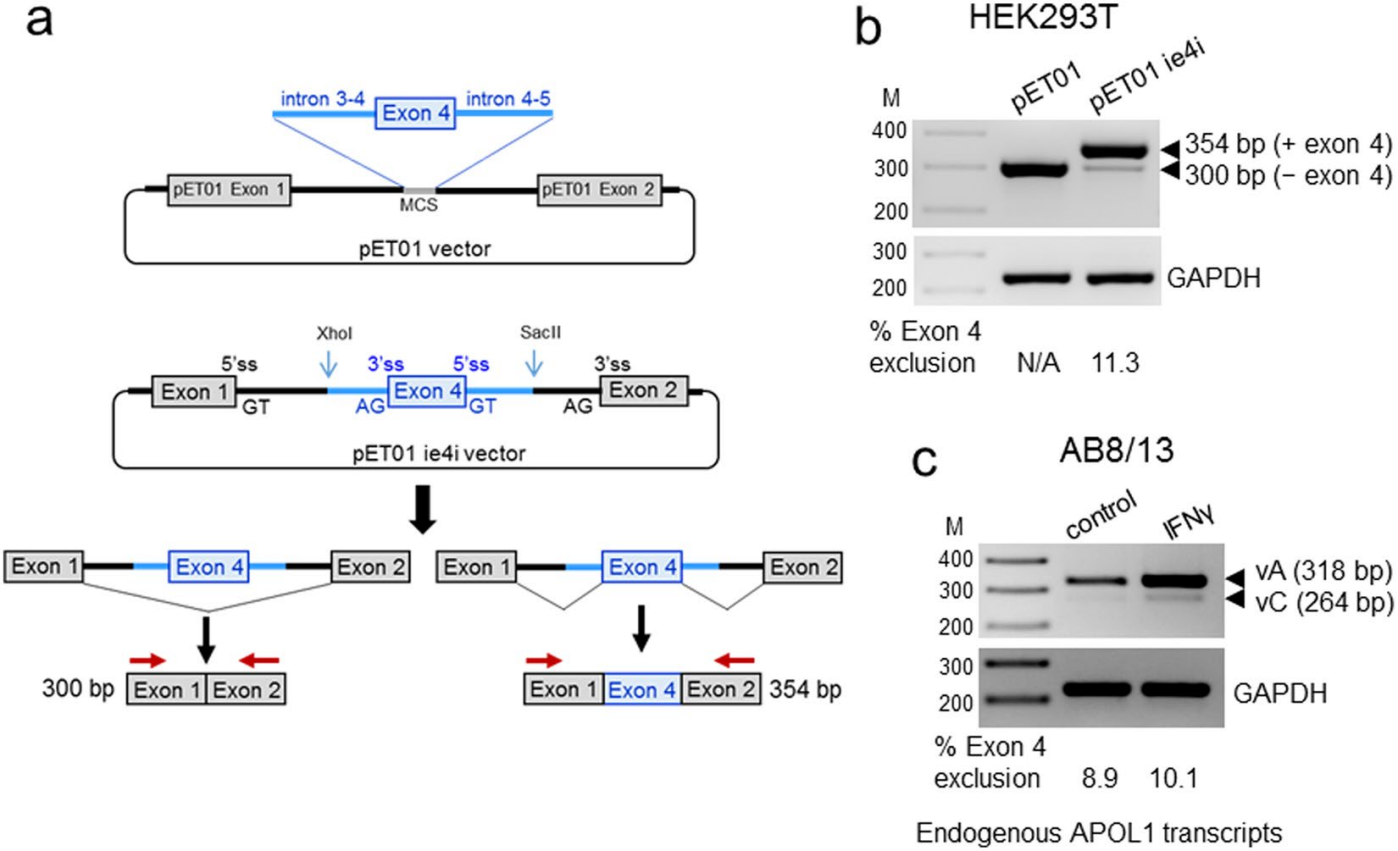
Generation of a splicing reporter pETie4i minigene for the analysis of the APOL1 exon 4 splicing. (**a**) APOL1 exon 4 and the flanking intron 3–4 sequence (includes 197 bp of full 2,083 bp) and a full intron 4–5 (182 bp), were PCR-amplified from genomic DNA of AB8/13 podocytes and cloned into the XhoI/SacII sites of the pET01 vector to create the pETie4i minigene construct. Two alternatively spliced transcripts can be produced by the pETie4i, transcript lacking exon 4 (300 bp) and with exon 4 (354 bp). MCS, multicloning site; ss, splice site. (**b**) pET01 and pETie4i constructs were transfected into HEK293T and 24 h later, total RNA was reverse transcribed to generate cDNA, which was subsequently amplified using a set of primers specific for pET01 exon 1 and exon 2 (red arrows) or primers specific for GAPDH (control). N/A, not applicable. (**c**) Endogenous APOL1 transcripts expressed in IFNγ-stimulated AB8/13 podocytes were analyzed by RT-PCR using a set of primers p1_3/p6 (see Fig. 1c) designed to amplify fragments of APOL1 vA and vC. The PCR products were separated on 2% agarose gel and stained with ethidium bromide. Predicted PCR product sizes (bp) are shown. Percentage of transcripts with or without exon 4 was calculated from densitometric scanning of the bands. Transcripts generated from transfected pETie4i (**b**) and endogenous APOL1 transcripts expressed in AB8/13 podocytes (**c**) show a similar pattern of exon 4 exclusion (11.3 vs.10.1%), indicating that pETie4i correctly reproduces the splicing pattern of endogenous APOL1 transcripts. M, DNA markers (bp). The gel images (**b** and **c)** were cropped. The full gel images are shown in Supplementary Fig. 2.

To identify trans-acting factors and cis-acting RNA elements that regulate exon 4 splicing, we generated an APOL1 splicing reporter minigene construct containing a full 54 base pair (bp) exon 4 and flanking intronic sequences amplified from the genomic DNA of AB8/13 podocytes, cloned into the exontrap vector pET01 (Fig. 2a). Due to the size of the intron between exons 3 and 4 (intron 3–4, 2083 bp), only 197 bp adjacent to exon 4 and the entire intron 4–5 (184 bp) were amplified and cloned into the pET01 vector. To analyze exon 4 splicing, an empty pET01 vector and pETie4i were transiently transfected into HEK293T cells and total RNA was extracted after 24 h then analyzed by RT-PCR. As expected, primers annealing to exon 1 and exon 2 of an empty pET01 vector amplified only a single RT-PCR product of 300 bp (Fig. 2b). The same primer pairs amplified two PCR products from pETie4i; a minor 300 bp product corresponding to the exon 4-free transcript and a major 354 bp product that included the 54 bp exon 4. The proportion of transcripts with and without exon 4 was calculated from densitometric intensities of the ethidium bromide stained PCR products. Results indicate that exon 4-lacking transcripts generated from the pETie4i minigene represented ~11% of amplified transcripts.

To investigate whether the splicing pattern of exon 4 in transcripts generated from a pETie4i minigene is similar to the splicing pattern of endogenous APOL1 transcripts expressed by IFNγ-stimulated AB8/13 podocytes, we used a set of primers that amplify fragments of APOL1 splice variants A and C (Fig. 1c). Figure 2c shows a very similar pattern of exon 4 splicing in AB8/13 podocytes with a strong retention (~90%) of exon 4 in endogenous transcripts. These results indicate that regulatory elements controlling exon 4 splicing are present in a cloned genomic DNA fragment encompassing a truncated intron 3–4, full exon 4, and intron 4–5. Thus, we conclude that our splicing minigene model correctly reproduces the splicing pattern of endogenous APOL1 transcripts and can be used to identify elements that may regulate exon 4 splicing.

### Identification of RNA splicing elements that regulate exon 4 splicing

**Figure 3:**
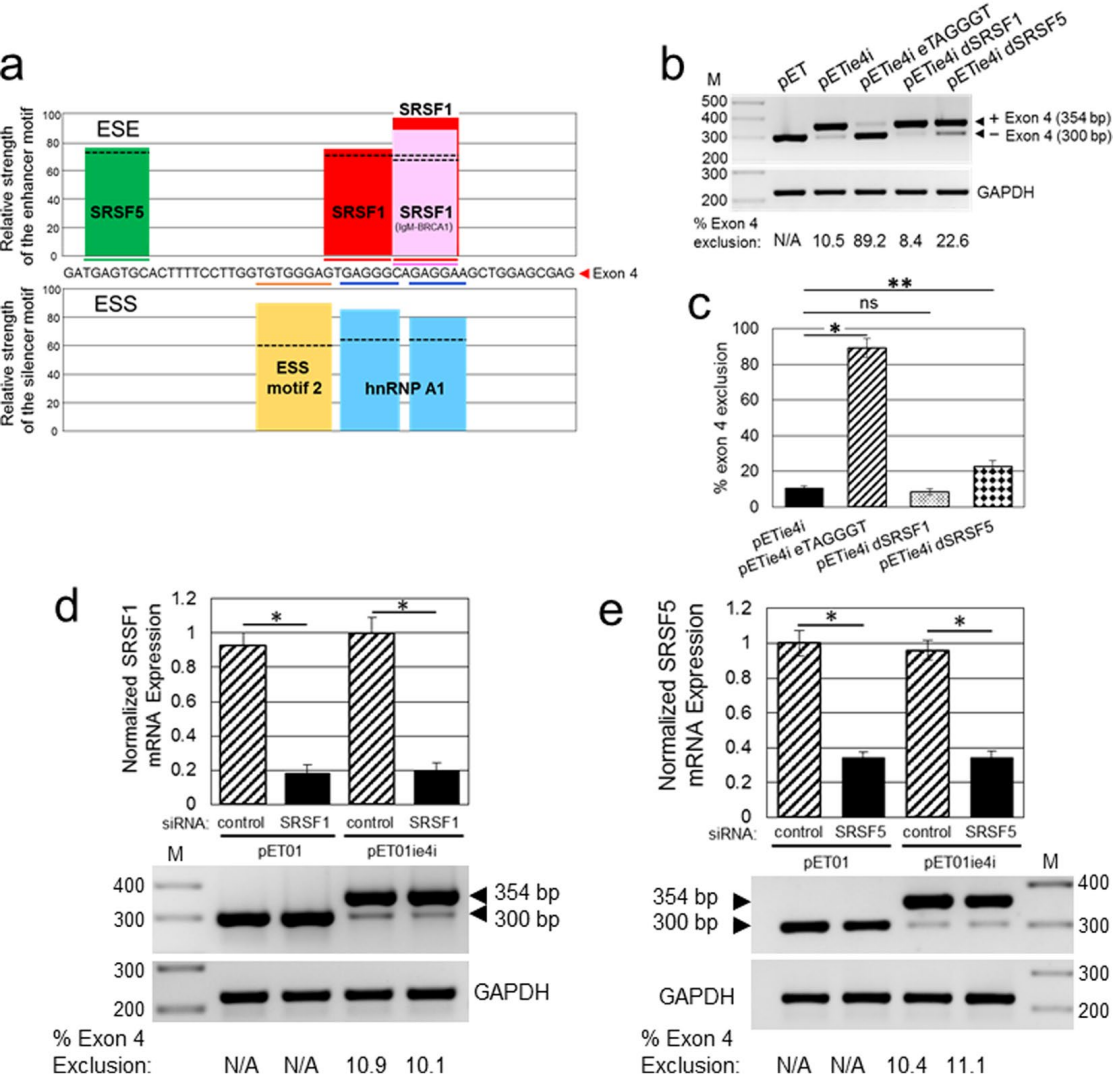
Effect of SRSF1 and SRSF5 on exon 4 skipping. (**a**) Identification of putative exonic splicing enhancer (ESE) and exonic splicing silencer (ESS) motifs in the APOL1 exon 4. The upper panel presents ESE motifs identified by ESEfinder 3.0. Scores above the threshold (black dotted lines) were found for SRSF1 and SRSF5 proteins. The lower panel shows ESS motifs predicted by Human Splicing Finder 3.0 (HSF3.0). Two major ESS motifs (ESS motif 2 and hnRNP A1) were identified. (**b**) Mutation in SRSF1 binding motif (GGCAG > ATATA, pETie4i dSRSF1), predicted by ESEfinder 3.0 to abolish SRSF1 binding motifs in exon 4, does not affect exon 4 splicing. Mutation in SRSF5 binding motif in exon 4 (TGAGTGC > TATATGC, pETie4i dSRSF5), which abolishes predicted by ESEfinder 3.0 binding motif for SRSF5, enhances moderately exon 4 skipping. Minigene with hnRNPA1 consensus motif in exon 4 (pETie4i eTAGGGT) served as positive control for exon 4 skipping. (**c**) Exon 4 exclusion was calculated from the intensities of RT-PCR bands shown in (b) and was expressed as a percentage of transcripts without exon 4 in a total pool of transcripts with and without exon 4 (100%). HEK293T cells were transfected with indicated minigene constructs and after 24 h, RNA was isolated and subjected to RT-PCR. Values are means ± SD of 3 independent experiments. *P < 0.001, **P < 0.005; ns, not statistically significant. pET, empty minigene control; pETie4i, minigene with exon 4 and adjacent introns. Effect of knockdown of SRSF1 (**d**) and SRSF5 (**e**) on exon 4 skipping. HEK293T cells were transfected with SRSF1 siRNA (**d**), SRSF5 siRNA (**e**) or control siRNA. After 24 h, the cells were transfected with pET01 or pETie4i minigenes and 24 h later total RNA was collected and subjected to RT-PCR. Values are means ± SD of 3 independent experiments. *P < 0.001. The gel images (**b**–**e**) were cropped. The full gel images are shown in Supplementary Fig. 3.

**Figure 4:**
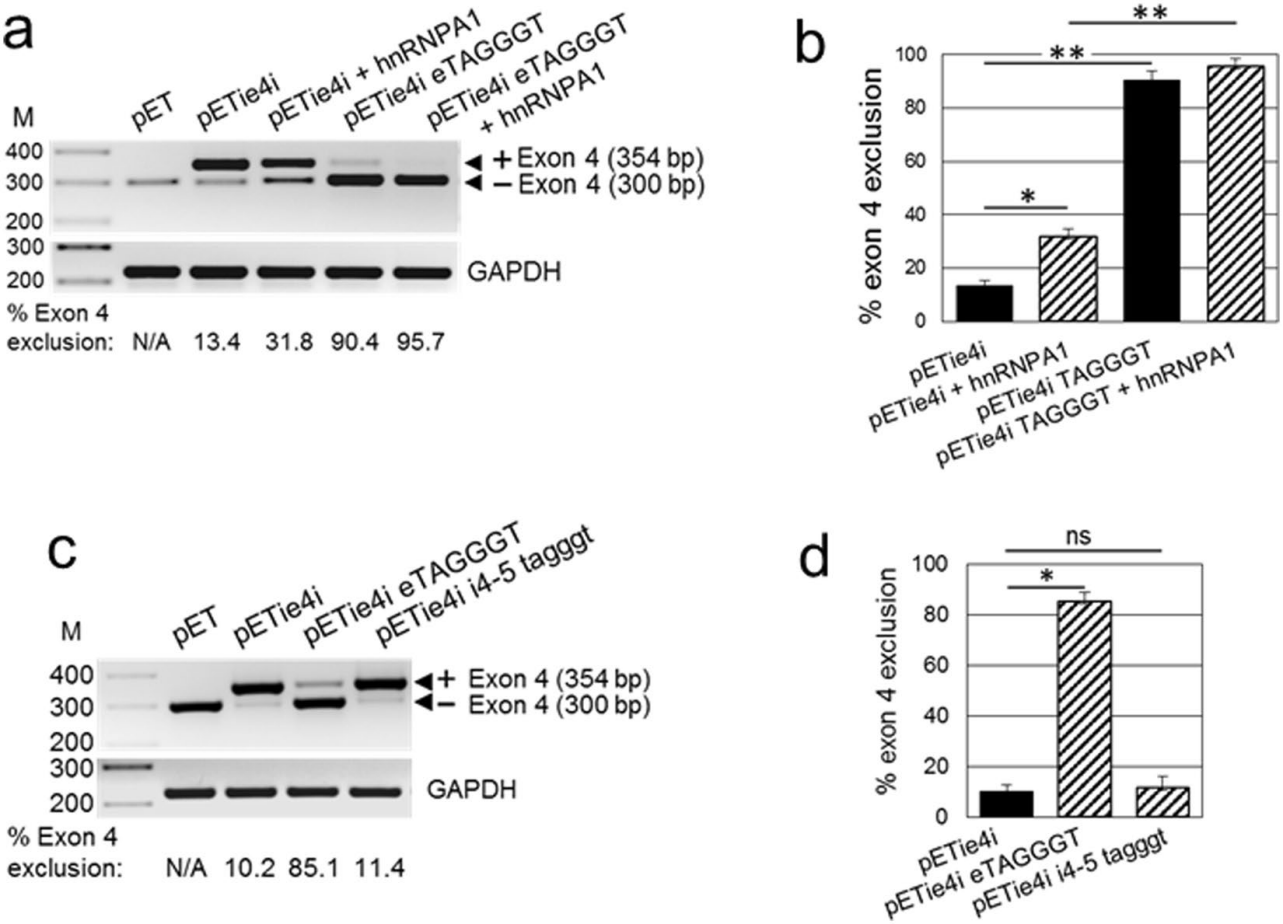
hnRNPA1 stimulates exon 4 skipping. (**a**) Exogenous hnRNPA1 increases exon 4 skipping from the pETie4i minigene transcript. AB8/13 podocytes were transfected with indicated pETie4i constructs in the absence or presence of hnRNPA1 expression vector. At 24 h post-transfection, isolated RNA was subjected to RT-PCR and exon 4 exclusion was analyzed. Generation of the hnRNPA1 consensus binding motif in exon 4 (GAGGGC > TAGGGT; pETie4i eTAGGGT) stimulates (90.4%) exon 4 skipping. This effect was further increased (95.7%) in the presence of exogenous hnRNPA1 (pETie4i eTAGGGT + hnRNPA1). (**b**) Exon 4 exclusion was calculated from the intensity of bands representing minigene transcripts with (354 bp) and without (300 bp) exon 4. Averages and SD from 3 independent experiments are shown. *P < 0.005, **P < 0.001. (**c**) Mutations that generate the hnRNPA1 consensus motif in intron 4–5 (pETie4i i4-5 tagggt) do not considerably affect exon 4 skipping. (**d**) Exon 4 exclusion was calculated as described in (**b**). HEK293T cells were transfected with indicated reporter minigene constructs and isolated RNA was subjected to RT-PCR analysis. Values are means ± SD of 3 independent experiments. *P < 0.001; ns, not statistically significant. The gel images (**a** and **d**) were cropped. The full gel images are shown in Supplementary Fig. 4.

To identify putative ESE motifs in exon 4, we used the web-based bioinformatics tool ESEfinder 3.0 that searches input sequences for high affinity binding sites for several serine/arginine-rich splicing factors (SRSF)^27^. ESEfinder predicted three clustered SRSF1 binding motifs and a single SRSF5 motif in exon 4 with scores above the threshold (Fig. 3a, top panel). The two highest score SRSF1 motifs (CAGAGGA) overlapped and could potentially promote inclusion of exon 4 in APOL1 transcripts. To investigate the possibility that SRSF1 binding motifs promote exon 4 inclusion, we mutated all 3 putative SRSF1 binding motifs by substituting a single pentamer GGCAG sequence with ATATA in a GTGAGGG/CAGAGGA motif (Fig. 3a,b, pETie4i dSRSF1). Although this substitution was predicted by ESEfinder to abolish binding to all 3 SRSF1 motifs in exon 4, it did not significantly affect exon 4 exclusion. In contrast, mutation TGAGTGC > TATATGC predicted to abolish the SRSF5 binding motif (Fig. 3b, pETie4i dSRSF5), slightly increased exclusion of exon 4 from the pETie4i transcripts. To rule out a low exclusion of exon 4 due to suboptimal pETie4i transcript recognition and splicing, we replaced an existing hnRNP A1 motif GAGGGC (Fig. 3a, bottom panel) with a consensus TAGGGT binding motif for hnRNPA1 (Fig. 3b, pETie4i eTAGGGT). The hnRNPA1 consensus motif increased exclusion of exon 4 to ~90% (Figs 3b,c and 4), indicating that pETie4i transcripts are correctly and efficiently recognized by the splicing machinery.

**Figure 5:**
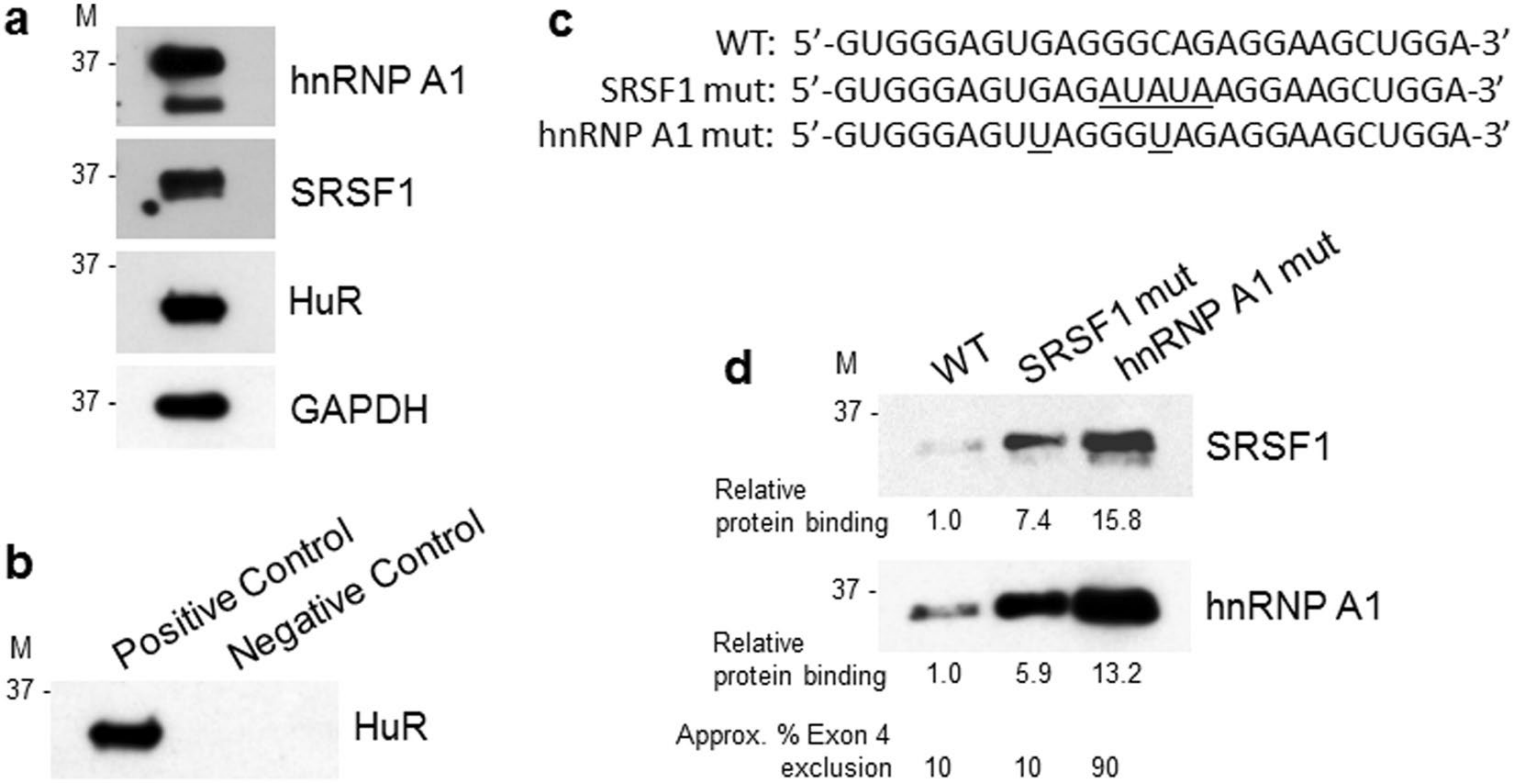
RNA pull-down analysis of SRSF1 and hnRNP A1 binding to their original and mutated RNA cis-acting motifs identified in exon 4. (**a**) HEK293T cells express hnRNP A1, SRSF1 and HuR proteins. Whole cell lysates used in RNA pull-down assays were analyzed by western blotting for the expression of endogenous hnRNP A1, SRSF1, HuR and GAPDH. Protein size markers (M) in kDa are indicated. (**b**) Immunoblotting analysis of HuR protein pulled down from HEK293T cell lysate using biotinylated fragment of the androgen receptor 3′-UTR RNA that contains HuR binding sites (Positive Control) or poly(A)_25_ RNA with no HuR binding sites (Negative Control). (**c**) Sequences of 3′-biotinylated RNA probes used for RNA pull-down assays. RNA probes encompass a 27-nucleotide fragment (nucleotides 23 to 49) of exon 4 that contain the putative RNA motifs for binding of SRSF1 and hnRNP A1 proteins (Fig. 3a). WT, a wild-type sequence contains predicted SRSF1 and hnRNP A1 binding sites; SRSF1 mut sequence contains mutations predicted by ESEfinder 3.0 to abolish or, as predicted by HSF 3.0, to reduce SRSF1 binding; hnRNP A1 mut represents RNA probe with hnRNP A1 consensus motif UAGGGU. Introduced mutations are underlined. (**d**) Immunoblotting analysis of SRSF1 and hnRNP A1 proteins pulled down using a wild-type (WT) and mutated SRSF1 and hnRNP A1 RNA probes described in (**c**). The biotinylated RNA probes were incubated with 100 μg of HEK293T cell lysate and bound proteins were separated by gel electrophoresis and immunoblotted for SRSF1 and hnRNP A1. The same exposure times for SRSF1 and hnRNP A1 are shown. Protein binding to mutated RNA probes was expressed relative to binding to the WT RNA probe and was calculated from the densitometric intensities of protein bands. Indicated approximate exon 4 exclusions from minigene transcripts are based on results shown in Figs 3 and 4. M, protein size markers in kDa. The gel images (**a**,**b** and **d**) were cropped. The full gel images are shown in Supplementary Fig. 5.

The effect of SRSF1 and SRSF5 binding proteins on regulation of exon 4 splicing was analyzed in HEK293T cells transfected with siRNA to reduce expression of endogenous SRSF1 and SRSF5 transcripts. Approximately 80% knockdown of SRSF1 mRNA (Fig. 5c) and ~65% knockdown of SRSF5 mRNA (Fig. 3e) in HEK293T cells did not affect the pattern of exon 4 splicing. A moderate increase of exon 4 exclusion with mutated SRSF5 binding site (Fig. 3b, pETie4i dSRSF5) indicates that 65% knockdown of SRSF5 mRNA may be insufficient to deplete SRSF5 protein below functional levels.

Since splicing efficiency of an exon is modulated by the interplay between splicing enhancers and silencers, we analyzed potential splicing silencer motifs in exon 4 and adjacent introns using Human Splicing Finder 3.0 (HSF 3.0). This program predicted several potential silencer motifs in exon 4 with scores above the threshold for ESS motif 2 and two binding motifs for heterogeneous nuclear ribonucleoproteins (hnRNPs) A1 (Fig. 3a, bottom panel). Since the hnRNP A1 motif often stimulates exon exclusion^28^, we investigated its effect on exon 4 splicing by modifying the original GAGGGC sequence of the highest score binding motif to match the hnRNPA1 consensus sequence TAGGGT. This mutation resulted in a dramatic increase in exon 4 exclusion from ~10% to ~90% (Fig. 4, pETie4i eTAGGGT), indicating that the low level of exon 4 exclusion results from a suboptimal original hnRNP A1 binding motif rather than from limiting levels of endogenous hnRNPA1 protein. Overexpression of hnRNPA1 protein had a significantly smaller impact on exon 4 skipping (Fig. 4a,b).

HSF3.0 also predicted the hnRNP A1 binding motif taggtt localized in intron 4–5 flanking exon 4. We examined the impact on exon 4 splicing of a single t > g mutation, creating a consensus hnRNPA1 motif tagggt in intron 4–5. In contrast to the hnRNPA1 consensus motif TAGGGT introduced in exon 4, the consensus motif generated in intron 4–5 failed to change exon 4 splicing (Fig. 4c,d). This suggests that the hnRNPA1 motif functions in a position-dependent manner, as reported for other motifs of the hnRNP family^29^. Our results suggest that constitutive splicing of exon 4 may result from a suboptimal hnRNPA1 binding motif.

### Inefficient binding of SRSF1 and hnRNP A1 to predicted RNA cis-acting motifs in exon 4

To confirm that suboptimal cis-acting RNA regulatory motifs are responsible for constitutive splicing of exon 4, we complemented our minigene-based splicing assays with the RNA pull-down analysis to evaluate binding of SRSF1 and hnRNP A1 proteins to their RNA cis-acting motifs identified by ESEfinder 3.0 or HSF 3.0 (Fig. 3a). RNA pull-down assays were performed using total HEK293T cell lysates and synthetic 27-nucleotide-long biotinylated RNA probes that span the putative SRSF1 and hnRNP A1 binding sites (Figs 3a and 5c). We have confirmed that endogenous SRSF1 and hnRNP A1 proteins are highly expressed in HEK293T cells (Fig. 5a). Control experiments verified that biotinylated RNA probe from the androgen receptor 3′-UTR that contains HuR binding motif specifically pulled down HuR protein. In contrast, poly(A)_25_ RNA probe without HuR motif showed no HuR protein binding (Fig. 5b). Although SRSF1 and hnRNP A1 proteins are abundantly expressed in HEK293T cells, the wild-type RNA probe showed very low binding of SRSF1 and slightly higher binding of hnRNP A1 (Fig. 5d). This contrasts with relatively high binding scores predicted by ESEfinder 3.0 and HSF 3.0 for SRSF1 and hnRNP A1 cis-acting motifs in exon 4. Curiously, mutations predicted by ESEfinder 3.0 to abolish SRSF1 binding to the SRSF1 mut probe considerably increased binding of both SRSF1 and hnRNP A1 (Fig. 5d). Increased binding of SRSF1 and hnRNP A1 did not however change exon 4 exclusion from the minigene transcripts (Fig. 3b,c). We speculate that generation of new SRSF1 and hnRNP A1 binding motifs by introduced mutations, as predicted by HSF 3.0, may partly explain this phenomenon. Substitution of a suboptimal GAGGGC hnRNP A1 binding motif with the consensus UAGGGU motif, strongly increased binding of hnRNP A1 and SRSF1 proteins to the probe. Importantly, binding of hnRNP A1 to its consensus motif in RNA probe produced ~90% exon 4 exclusion, as determined in minigene assays, and was not affected by SRSF1 binding (Fig. 4).

### Morpholino oligonucleotide targeting exon 4 donor splice site stimulates alternative splicing and APOL1 protein isoform switch

**Figure 6:**
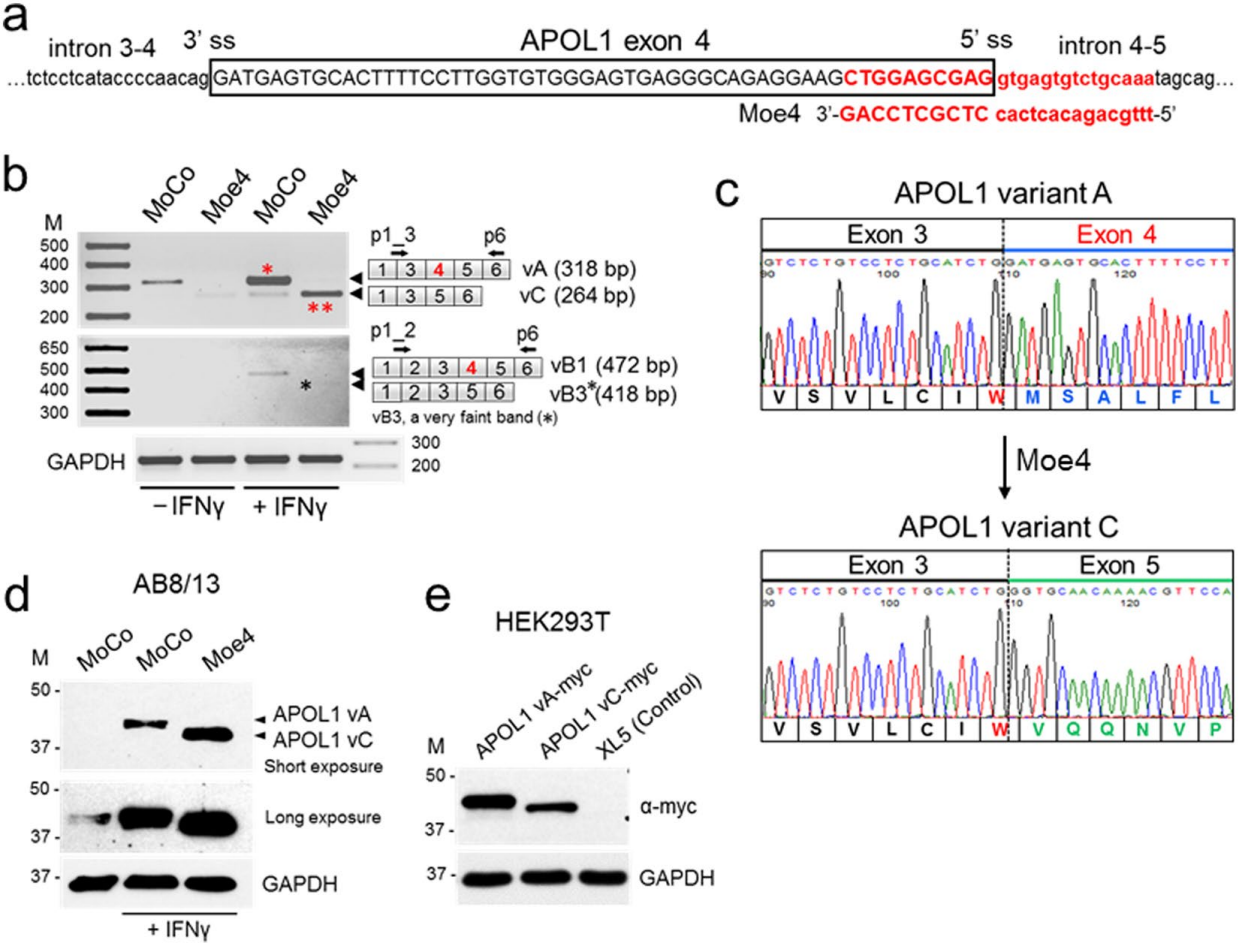
Morpholino oligonucleotide Moe4 promotes exclusion of exon 4 from endogenous APOL1 transcripts and causes APOL1 protein variant switch. (**a**) Annealing positions and nucleotide sequence of morpholino Moe4 that blocks the 5′ splice site (5′ss) of exon 4 is shown. (**b**) Moe4 changes a splicing pattern of endogenous APOL1 exon 4 in podocytes. AB8/13 podocytes were treated for 24 h with IFNγ (50 ng/ml) to stimulate expression of APOL1 transcripts followed by the exposure for 24 h to Moe4 or control morpholino (MoCo). Total RNA was analyzed by RT-PCR for the expression of APOL1 splice variants using indicated set of primers. Amplified PCR products were resolved on 2% agarose gel. Predicted sizes of amplicons corresponding to APOL1 splice variants are shown. (**c**) To confirm identity of the APOL1 splice variants, DNA bands representing vA (*) and vC (**) variants were cloned and sequenced. Fragments of the amino acid sequences of the exon 4-positive vA and Moe4-induced exon 4-negative vC were determined from DNA sequences. Note that tryptophan residue (W, red) is encoded by two last nucleotides (TG) of exon 3 and the first nucleotide (G) of the exon 4 or exon 5. Thus, alternative splicing of exon 4 does not change the reading frame. (**d**) Moe4 promotes APOL1 protein variant switch. Protein lysates collected from AB8/13 podocytes treated as described in (**b**) were analyzed by western blotting for the expression of APOL1 and GAPDH (control). APOL1 vC (isoform lacking the exon 4-encoded sequence) migrates faster than vA protein (isoform with exon 4-encoded sequence). (**e**) Transient transfection of APOL1-negative HEK293T cells with myc-tagged APOL1 vA and APOL1 vC confirms that both isoforms migrate in a SDS gel as proteins of different sizes. XL5, pCMV6-XL5 empty vector. M, molecular weight markers (kDa). The gel images (**d**,**e**) were cropped. The full gel images are shown in Supplementary Fig. 6.

A limited role for cis-acting elements in regulation of exon 4 splicing prompted us to induce exon 4 skipping by targeting the exon 4 splice sites with antisense morpholino oligonucleotides. Only one morpholino (Fig. 6a, Moe4) complementary to the exon 4/intron 4–5 region encompassing the 5′ss fulfilled the criteria for effective antisense morpholino^30^. To test whether blocking the 5′ss by Moe4 changes the splicing pattern of endogenous APOL1 transcripts, AB8/13 podocytes were pretreated for 24 h with Moe4 or non-targeting standard control morpholino (MoCo) followed by activation with IFNγ to increase expression of APOL1 transcripts. Results presented in Fig. 6b show that exon 4-positive transcript vA is a major transcript expressed by AB8/13 podocytes. Similarly, treatment with control morpholino (MoCo) did not change the pattern of exon 4 splicing. In contrast, treatment with Moe4 resulted in almost complete exclusion of exon 4 from endogenous APOL1 transcripts with vC representing the major splice variant expressed in podocytes. Exon 4-positive vB1 was expressed at low level (<5% total APOL1 transcripts) after IFNγ stimulation. Exon 4-negative vB3 was barely detectable after Moe4 treatment (Fig. 6b, black star). The identity of PCR amplicons corresponding to vA (318 bp) and vC (264 bp) was confirmed by DNA sequencing (Fig. 6c) of DNA amplicons isolated from the gel (Fig. 6b, red stars).

To investigate whether Moe4-induced alternatively spliced transcripts are functional, protein lysates were prepared from AB8/13 podocytes treated with Moe4 or control MoCo. Figure 6d shows that treatment with Moe4 induced expression of APOL1 protein (vC), which migrated faster than variant A due to exclusion of the 18-amino acid sequence encoded by exon 4 (~1.8 kDa). This endogenous APOL1 vC protein migrated on a gel similarly to exogenous APOL1 vC expressed in HEK293T cells (Fig. 6e). These experiments indicate that exclusion of exon 4 from endogenous APOL1 transcripts can be efficiently induced by antisense morpholino oligonucleotides targeting the 5′ss of exon 4.

## Discussion

The *APOL* gene family is composed of six genes located on human chromosome 22 and is involved in innate immunity^25,26,31–33^. Out of all the APOL family proteins, APOL1 is the only member that contains a signal peptide that allows for its secretion into blood to provide immunity against *T. brucei*^25,26,31–34^. The other APOL family members, which do not contain a signal peptide, are mainly localized to the endoplasmic reticulum and the cytoplasm^25,31–33^. Exon 4 encodes a part of the N-terminal sequence that constitutes a signal peptide necessary for proper intracellular targeting and secretion of APOL1. This suggests that constitutive splicing of exon 4 may be necessary for efficient secretion of APOL1 through the classical secretory pathway. Commonly used SignalP 4.0 computational tool^35^ predicts a very strong signal peptide in exon 4-positive variant A and a very weak signal peptide in variant C, suggesting that variant C could be potentially less efficiently secreted. Whether the truncated signal peptide affects secretion of variant C *in vivo* and/or modifies its interaction with factors known to promote or mediate the kidney disease phenotype^36–38^ is presently unknown. Here, we provide evidence that APOL1 splice variant A (vA) with exon 4 is a major transcript expressed in kidney glomerular and tubular cells stimulated with IFNγ. Transcripts lacking exon 4 (referred here as variant C; vC) represent only a small fraction of APOL1 transcripts expressed in the kidney (Fig. 1). A similar pattern of APOL1 transcript expression has also been demonstrated in human coronary artery endothelial cells^39^.

Alternative splicing can generate multiple mRNAs and proteins from a single primary transcript. This prevalent process greatly expands the coding capacity of complex genomes because it can generate products with different, even antagonistic, properties from a single gene locus^22^. The APOL1 gene is composed of seven exons and produces several alternatively spliced transcripts that may differentially include exons 2, 3, and 4^24–26^. Exons 3 and 4 encode a part of the N-terminal sequence that constitutes a signal peptide necessary for secretion of APOL1 variant A. Since exon 4 encodes the C-terminal part of the signal peptide, exclusion of exon 4 may affect functionality of a truncated signal peptide and affect secretion of APOL1.

APOL1 expression and additional cofactors or second hits are required for development of a disease phenotype^16,40^. Recently, a soluble urokinase plasminogen activator receptor (suPAR) was shown to synergize with the APOL1 risk variants G1 and G2 to activate αvβ3 integrin on podocytes^18^. These data suggest that, for the podocyte injury to occur, the secreted APOL1 risk variant proteins need to be internalized by the cell in a urokinase receptor and β3-integrin-dependent manner. In this context, we speculate that the development of APOL1-related kidney disease could be mitigated even in the presence of risk alleles by preventing the formation of the tripartite complex. Thus, to address this question, it would be informative to generate a mouse model with inducible expression of APOL1 splice variant C with the wild-type and risk alleles, using a recently described approach^4^. Since modification of APOL1 signal peptide could potentially lead to expression of APOL1 isoform with different biological properties, we investigated the mechanism that controls alternative splicing of exon 4.

To identify the elements that regulate exon 4 splicing, we generated an APOL1 splicing reporter minigene construct pETie4i containing a full 54 bp exon 4 and flanking intronic sequences (Fig. 2a). Because of the size of the intron located between exons 3 and 4 (intron 3–4, 2083 bp), only 197 bp adjacent to exon 4 and the entire intron 4–5 (184 bp) were amplified and cloned into the pET01 vector. Validation experiments confirmed that the splicing pattern of exon 4 from the pETie4i transcript was similar to the splicing pattern of endogenous APOL1 transcripts expressed in podocytes stimulated with IFNγ (Fig. 2b,c). Although in some cases cell-specific regulatory elements may be located far away from the exon, regulatory elements are usually found within 300 nucleotides upstream and downstream of the exon^41–43^. Transcription-coupled alternative splicing supports this model^44,45^. Based on our results, we conclude that a simplified minigene reporter construct can be used to identify molecular elements controlling exon 4 splicing.

Using bioinformatics tools ESEfinder and HSF3.0, we identified in exon 4 putative ESE elements, SRSF5 and three SRSF1 motifs, and ESS elements, ESS motif 2 and two adjacent hnRNP A1 motifs (Fig. 3a). Using minigene analysis we show here that mutations disrupting predicted SRSF1 binding motifs in exon 4, as well as siRNA-mediated downregulation of SRSF1 transcript, did not increase exon 4 exclusion. This indicates that SRSF1 motifs are non-functional in exon 4 (Fig. 3). A small but statistically significant increase in exon 4 exclusion was detected after disrupting a predicted SRSF5 motif (Fig. 4a,b). However, SRSF5 siRNA did not affect exon 4 splicing (Fig. 4d), suggesting that 65% downregulation of SRSF5 mRNA may be insufficient to detect a small effect on exon 4 splicing.

Overlap with two hnRNP A1 motifs could explain the lack of SRSF1 binding motif effect on exon 4 splicing (Fig. 3a). The hnRNP A1 motifs often inhibit inclusion of an exon, when engaged by hnRNP A1 proteins^28^, by sterically blocking access of SRSF1 proteins to their cognate motifs. We observe a small but statistically significant exclusion of exon 4 in cells overexpressing hnRNPA1 proteins (Fig. 4a,b). This moderate effect on exon 4 exclusion was potently increased by replacing the original hnRNP A1 motif GAGGGC with a consensus binding motif TAGGGT, indicating that original hnRNP A1 motifs are suboptimal for inducing significant changes in exon 4 splicing. We identified a putative hnRNP A1 motif in intron 4–5 adjacent to exon 4, which we modified to reflect hnRNP A1 consensus motif (Fig. 4c,d). These changes did not affect exon 4 splicing, indicating that in the context of exon 4, hnRNP A1 motif may function in a position-dependent manner. In this regard, position-dependent splicing mediated by hnRNP or SRSF proteins has been documented^46^. Given that hnRNP A1 protein is one of the most abundant and ubiquitously expressed members of the hnRNP family, and is engaged in many aspects of gene expression, splicing, and stability as well as microRNA processing and telomere maintenance^46^, overexpression of this factor does not seem a reasonable approach to increase exon 4 exclusion from APOL1 transcripts. In summary, we identified several cis-acting regulatory motifs that could potentially regulate exon 4 splicing. The lack of an optimal consensus hnRNP A1 motif in exon 4 may explain the robust inclusion of this exon in APOL1 transcripts and expression of the signal peptide in major APOL1 protein isoforms. Together, our results indicate that SRSF1 binding does not modify exon 4 splicing and that suboptimal hnRNP A1 cis-elements located in exon 4 are not sufficient to stimulate exon 4 exclusion from the minigene or endogenous APOL1 transcripts. This conclusion is supported by our results indicating that exon 4 is constitutively spliced in major APOL1 variant A expressed by glomerular and extraglomerular cells (Fig. 1).

To promote deletion of exon 4 from APOL1 transcripts, we tested antisense morpholino oligonucleotides targeting the exon 4/intron boundaries. Only one morpholino targeting the 5′ss (Moe4) fulfilled criteria for an effective splice-switching morpholino^30^ so was used in experiments. Since binding of a small nuclear ribonucleoprotein complex U1 to 5′ss is the first step in spliceosome assembly^47^, blocking the 5′ss by Moe4 could potentially change the splicing pattern of endogenous APOL1 transcripts. Indeed, RT-PCR analysis indicate that Moe4, but not control morpholino MoCo, effectively induces exon 4 exclusion from endogenous APOL1 transcripts expressed by podocytes stimulated with IFNγ (Fig. 6b). The identity of PCR amplicons corresponding to spliced isoforms vA and vC was confirmed by DNA sequencing of respective DNA products isolated from the gel (Fig. 6c). Alternatively spliced transcript lacking exon 4 was still functional; treatment with Moe4 induced expression of APOL1 vC protein predictably migrated faster than vA on a gel due to lack of an 18-amino acid sequence (~1.8 kDa) encoded by exon 4 (Fig. 6d). Comparably, APOL1 vC protein expressed from a construct transfected in APOL1-negative HEK293T cells migrated faster than APOL1 vA expressed from the transfected construct (Fig. 6e).

In conclusion, our study indicates that exon 4 is constitutively spliced in APOL1 transcripts expressed in kidney cells. Exclusion of exon 4 from endogenous APOL1 transcripts by morpholino Moe4 blocks the 5′ss of exon 4, resulting in the expression of non-toxic APOL1 proteins independent of the presence of risk alleles. APOL1 secretion may form a tripartite complex with suPAR and αvβ3 integrin, leading to chronic kidney disease^18^. Therefore, changing the splicing pattern of APOL1 risk variants to delete exon 4 may impair APOL1 secretion, prevent formation of the tripartite complex, and possibly mitigate the APOL1 kidney disease phenotype.

## Methods

### Cell culture and transient transfection

HEK293T cells were maintained at 37 °C in DMEM medium supplemented with 10% fetal bovine serum and gentamycin (50 μg/ml). Cells were seeded on collagen-coated 6-well plates (5.5 × 10^5^ cells/ml) and transfected with 2 μg of total DNA per well using polyethylenimine (PEI, 25,000 mol wt, Polysciences).

AB8/13 podocytes were cultured at 33 °C in RPMI 1640 media supplemented with insulin-transferrin-selenium (ITS, Invitrogen), gentamycin (50 μg/ml), and 10% FBS (Clontech). For a transient transfection, AB8/13 podocytes were cultivated at 33 °C in 6-well plates (3.2 × 10^5^ cells/well) and transfected with 2 μg of total DNA per well using jetPrime reagent (VWR).

### APOL1 expression vectors

APOL1 cDNA corresponding to splice variant A (NM_003661) was obtained in pCMV6-XL5 vector (OriGene). The open reading frame of APOL1 genes expressed from pCMV6-XL5 was modified by PCR with myc epitope^24^. APOL1 deletion mutants were created using the QuickChange II site-directed mutagenesis kit (Stratagene). APOL1 variant C (NM_001136541) was generated by deletion of 54 nucleotides corresponding to the full exon 4 of APOL1 variant A using a set of two complementary primers: 5′-GAGTCTCTGTC CTCTGCATCTGGGTGCAACAAAACGTTCCAAGTGGG-3′ and 5′-CCCACTTGGAACGTTTTG TTGCACCCAGATGCAGAGGACAGAGACTC-3′. Integrity of APOL1 constructs was confirmed by DNA sequencing.

### Generation of a splicing reporter minigene

APOL1 exon 4 and the flanking intron 3–4 sequence (includes 197 bp of full 2,083 bp) and full intron 4–5 (182 bp), were amplified by PCR using DNA isolated from human glomerular podocytes AB8/13 and the following primers as a template: XhoI (underlined) forward 5′-ATATATATCTCGAGACATGTAAGAGGCTCTGC-3′ and SacII (underlined) reverse 5′-ATATATATCCGCGGCTTGAGGAGGAGAAAAC-3′. The APOL1 sequences were obtained from the Ensembl genome browser. The selection of 197 nucleotides from the intron 3–4 was based on analysis of SNPs present upstream of 197 nucleotides that could potentially affect annealing of PCR primers designed to amplify this genomic DNA fragment. To avoid this possibility, we designed the intron 3–4 PCR primer that anneals to a known SNP-free fragment. Amplicons were cloned into the XhoI/SacII sites of the pET01 vector (MoBiTec, Germany) to create the pETie4i minigene construct. All constructs were verified by DNA sequencing.

### Analysis of a minigene pETie4i splicing

Total RNA was isolated from HEK293T cells or AB8/13 podocytes transfected with pETie4i constructs. RNA was purified using Quick RNA MiniPrep kit (Zymo Research) and additionally treated with DNase using TURBO DNA-free kit (Ambion) to remove residual contaminating DNA. RNA was reverse-transcribed using iScript cDNA synthesis kit (Bio-Rad), and cDNA was subjected to PCR using Platinum *Taq* DNA polymerase (Life Technologies) using cycling conditions: 95 °C for 1 min followed by 30 cycles of 95 °C for 30 s, 60 °C for 30 s, and 72 °C for 45 s. Sequences of PCR primers (250 nM) were as follows: pET01 forward: 5′-CAGGATCGATCTGCTTCCTG-3′, pET01 reverse: 5′-GTTGGTAGAGAGAGCAGATG-3′, GAPDH forward: 5′-GAAGGTGAAGGTCGGAGT-3′, GAPDH reverse: 5′-GAAGATGGTGATGGGATTTC-3′. PCR products were separated on 2% agarose gel and visualized with ethidium bromide.

### Analysis of endogenous APOL1 splice variant transcripts in human podocytes

Total RNA was purified from AB8/13 podocytes and processed as described above. Primers p1_3 and p6 were used to amplify fragments of APOL1 variants A and C, and p1_2 and p6 were used for amplification of APOL1 variants B1 and B3. The sequences of primers were as follows: p1_3: 5′-GGAAGATTCCTTGGAGGAGGC-3′, p6: 5′-CGTTCCAGGCCTCATTATCA-3′, p1_2: 5′-GGAAGATTCCTTGACTTCTGGGGT-3′. GAPDH was amplified with primers described above. PCR products were resolved on 2% agarose gel and visualized with ethidium bromide.

The predicted sizes of PCR amplicons were as follows: APOL1 vA - 318 bp, vC - 264 bp, vB1 - 472 bp and vB3 - 418 bp. For DNA sequencing analysis, DNA bands were excised and cloned directly into pJET vector using CloneJET PCR cloning kit (Thermo Scientific) as described^24^.

### Densitometric Analysis

The relative amounts of PCR products were quantitated from the intensity of DNA bands using Quantity One software (Bio-Rad). Percentage of transcripts including (A) and excluding (B) exon 4 were assessed and the percentage of exon 4 exclusion was calculated using the formula: 100 × [B/(A + B)]. Percentage of transcripts without exon 4 (vC %) was calculated from densitometric scanning of the vA and vC band intensities (vC % = [vC/vA + vC] × 100).

### RNA pull-down assay

RNA probes were synthesized by Integrated DNA Technologies. To ensure that RNA labeling will not compromise RNA structure or interfere with protein-RNA interactions, RNA probes were ligated at the 3′ end with desthiobiotinylated cytidine bisphosphate using a Pierce RNA 3′ end desthiobiotinylation kit (Thermo Scientific) according to the manufacturer’s protocol. Biotinylated RNA probes (60 pmol) were bound to streptavidin magnetic beads and incubated for 1 h on ice with 100 μg of HEK293T total protein lysate prepared in a buffer containing 25 mM Tris pH 7.4, 150 mM NaCl, 1% NP40, 1 mM EDTA, 5% glycerol and proteinase inhibitor cocktail (Complete Ultra, Roche). The RNA-protein complexes were isolated using a Pierce magnetic RNA-protein pull-down kit (Thermo Scientific) according to the manufacturer’s protocol. RNA-bound proteins were eluted by boiling for 10 min in 2 × SDS loading buffer and resolved on a 10% SDS-PAGE. RNA-bound proteins were detected using anti-SRSF1 (SF2/ASF, Santa Cruz, sc-33652, 1:500), anti-hnRNP A1 (Santa Cruz, sc-56700, 1:500) and anti-HuR (Thermo Scientific, #1862775, 1:1000). The sequences of the RNA probes that encompass 27-nucleotide-long fragments (nucleotides 23 to 49) of a 54-nucleotide long exon 4 were as follows: wild-type: 5′-GUGGGAGUGAGGGCAGAGGAAGCUGGA-3′; SRSF1 mut: 5′-GUGGGAGUGAGAUAUAAGGAAGCUGGA-3′; hnRNP A1 mut: 5′-GUGGGAGUUAGGGUAGAGGAAGCUGGA-3′.

A control pull-down assay was performed using the 3′ untranslated region (UTR) of androgen receptor RNA (3′-UTR RNA) and poly(A)_25_ RNA (both provided with the kit, Thermo Scientific). Both RNA probes were 3′ biotinylated and used as positive and negative controls, respectively. HuR protein that specifically binds to the 3′-UTR RNA of androgen receptor was detected by immunoblotting using anti-HuR antibodies provided with the kit (Thermo Scientific).

### Western blot analysis

Total cell extracts were harvested in RIPA buffer with protease inhibitor cocktail (Complete Ultra, Roche). Cell lysates (20–25 μg protein/lane) were resolved on 10% SDS-PAGE, transferred to a nitrocellulose membrane, and analyzed for the expression of endogenous APOL1 (Sigma, HPA018885, 1:5000), transfected APOL1-myc (Bethyl, anti-myc A190-105A, 1:10000), and GAPDH (Santa Cruz, sc-25778, 1:3000) using the WesternBright ECL system (Advansta).

### Computational analysis of splicing factor binding sites

The *in silico* splicing analyses were performed using bioinformatics tools Human Splicing Finder 3.0 (HSF 3.0) and ESEfinder 3.0.

### Morpholino oligonucleotides

A 25-mer morpholino oligonucleotide complementary to the exon 4/intron 4–5 junction and encompassing the 5′ss (Moe4, 5′-TTTGCAGACACTCACCTCGCTCCAG-3′) or a standard control morpholino (MoCo, 5′-CCTCCTACCTCAGTTACAATTTATA-3′) were designed and synthesized by Gene Tools (Philomath, OR). Morpholinos were synthesized as octaguanidine dendrimer conjugates (Vivo-morpholinos) to allow for cell entry without need for transfection reagent. AB8/13 podocytes were pretreated with 10 μM Moe4 or MoCo and after 24 h the cells were left untreated or were incubated for 24 h with human IFNγ (50 ng/ml, Peprotech). Both total RNA and proteins were collected. RNA was reverse transcribed using iScript cDNA synthesis kit (Bio-Rad) and generated cDNA was subjected to PCR. Transcripts representing APOL1 variant A (vA) and vC were amplified by PCR using a set of primers: p1_3 [GGAAGATTCCTTGGAGGAGGC] and p6 [CGTTCCAGGCCTCATTATCA]. Primer set p1_2 [GGAAGATTCCTTGACTTCTGGGGT] and p6 were designed to specifically amplify APOL1 vB1 and vB3. P1_3 and p1_2 denote primers that hybridize to a junction between exon 1 and 3, and exon 1 and 2, respectively. PCR products were resolved on 1.5% agarose gel and visualized with ethidium bromide. DNA bands representing 318 bp and 264 bp transcript fragments were excised from the gel, extracted, cloned directly into pJET vector, and sequenced^24^. DNA sequencing confirmed inclusion of exon 4 in 318 bp amplicon and lack of exon 4 in 264 bp amplicon.

### Statistical analysis

All experiments were repeated at least three times on independently prepared samples. Data are presented as means ± SD (standard deviation). For comparison of mean values between two groups, a Student’s *t*-test was performed. A difference was considered significant if the *P* value was less than 0.05.

### Data availability statement

The datasets generated during and/or analyzed during the current study are available from the corresponding author on reasonable request.

## Electronic supplementary material

Supplementary Information

## References

[1] Genovese G (2010). Association of trypanolytic ApoL1 variants with kidney disease in African Americans. Science.

[2] Tzur S (2010). Missense mutations in the APOL1 gene are highly associated with end stage kidney disease risk previously attributed to the MYH9 gene. Hum Genet.

[3] Perez-Morga D (2005). Apolipoprotein L-I promotes trypanosome lysis by forming pores in lysosomal membranes. Science.

[4] Beckerman P (2017). Transgenic expression of human APOL1 risk variants in podocytes induces kidney disease in mice. Nat Med.

[5] Parsa A (2013). APOL1 risk variants, race, and progression of chronic kidney disease. N Engl J Med.

[6] Cheng D (2015). Biogenesis and cytotoxicity of APOL1 renal risk variant proteins in hepatocytes and hepatoma cells. J Lipid Res.

[7] Lan X (2014). APOL1 risk variants enhance podocyte necrosis through compromising lysosomal membrane permeability. Am J Physiol Renal Physiol.

[8] Lan X (2015). Protein domains of APOL1 and its risk variants. Exp Mol Pathol.

[9] Thomson R (2014). Evolution of the primate trypanolytic factor APOL1. Proc Natl Acad Sci USA.

[10] Wan G (2008). Apolipoprotein L1, a novel Bcl-2 homology domain 3-only lipid-binding protein, induces autophagic cell death. J Biol Chem.

[11] Zhaorigetu S, Wan G, Kaini R, Jiang Z, Hu CA (2008). ApoL1, a BH3-only lipid-binding protein, induces autophagic cell death. Autophagy.

[12] Olabisi OA (2016). APOL1 kidney disease risk variants cause cytotoxicity by depleting cellular potassium and inducing stress-activated protein kinases. Proc Natl Acad Sci USA.

[13] Kruzel-Davila E (2017). APOL1-mediated cell injury involves disruption of conserved trafficking processes. J Am Soc Nephrol.

[14] Granado D (2017). Intracellular APOL1 risk variants cause cytotoxicity accompanied by energy depletion. J Am Soc Nephrol.

[15] Ma L (2017). APOL1 renal-risk variants induce mitochondrial dysfunction. J Am Soc Nephrol.

[16] Freedman BI, Skorecki K (2014). Gene-gene and gene-environment interactions in apolipoprotein L1 gene-associated nephropathy. Clin J Am Soc Nephrol.

[17] Hayek SS (2015). Soluble urokinase receptor and chronic kidney disease. N Engl J Med.

[18] Hayek SS (2017). A tripartite complex of suPAR, APOL1 risk variants and αvβ3 integrin on podocytes mediates chronic kidney disease. Nat Med.

[19] Chen M, Manley JL (2009). Mechanisms of alternative splicing regulation: insights from molecular and genomics approaches. Nat Rev Mol Cell Biol.

[20] Graveley BR (2000). Sorting out the complexity of SR protein functions. RNA.

[21] Long JC, Caceres JF (2009). The SR protein family of splicing factors: master regulators of gene expression. Biochem J.

[22] Wang Z, Burge CB (2008). Splicing regulation: from a parts list of regulatory elements to an integrated splicing code. RNA.

[23] Wang Z, Xiao X, Van Nostrand E, Burge CB (2006). General and specific functions of exonic splicing silencers in splicing control. Mol Cell.

[24] Khatua AK (2015). Exon 4-encoded sequence is a major determinant of cytotoxicity of apolipoprotein L1. Am J Physiol Cell Physiol.

[25] Duchateau PN, Pullinger CR, Cho MH, Eng C, Kane JP (2001). Apolipoprotein L gene family: tissue-specific expression, splicing, promoter regions; discovery of a new gene. J Lipid Res.

[26] Smith EE, Malik HS (2009). The apolipoprotein L family of programmed cell death and immunity genes rapidly evolved in primates at discrete sites of host-pathogen interactions. Genome Res.

[27] Cartegni L, Wang J, Zhu Z, Zhang MQ, Krainer AR (2003). ESEfinder: A web resource to identify exonic splicing enhancers. Nucleic Acids Res.

[28] Talukdar I (2011). hnRNP A1 and hnRNP F modulate the alternative splicing of exon 11 of the insulin receptor gene. PLoS One.

[29] Fu XD, Ares M (2014). Context-dependent control of alternative splicing by RNA-binding proteins. Nat Rev Genet.

[30] Moulton, J. D. Using morpholinos to control gene expression. *Curr Protoc Nucleic Acid Chem* **68**, 4 30 31–34 30 29, 10.1002/cpnc.21 (2017). 10.1002/cpnc.21 PMC7162182 28252184

[31] Page NM, Butlin DJ, Lomthaisong K, Lowry PJ (2001). The human apolipoprotein L gene cluster: identification, classification, and sites of distribution. Genomics.

[32] Duchateau PN (2000). Plasma apolipoprotein L concentrations correlate with plasma triglycerides and cholesterol levels in normolipidemic, hyperlipidemic, and diabetic subjects. J Lipid Res.

[33] Duchateau PN (1997). Apolipoprotein L, a new human high density lipoprotein apolipoprotein expressed by the pancreas. Identification, cloning, characterization, and plasma distribution of apolipoprotein L. J Biol Chem.

[34] Vanwalleghem G (2015). Coupling of lysosomal and mitochondrial membrane permeabilization in trypanolysis by APOL1. Nat Commun.

[35] Petersen TN, Brunak S, von Heijne G, Nielsen H (2011). SignalP 4.0: discriminating signal peptides from transmembrane regions. Nat Methods.

[36] Hahm E (2017). Bone marrow-derived immature myeloid cells are a main source of circulating suPAR contributing to proteinuric kidney disease. Nat Med.

[37] Hayek SS (2017). A tripartite complex of suPAR, APOL1 risk variants and alphavbeta3 integrin on podocytes mediates chronic kidney disease. Nat Med.

[38] Wei C (2011). Circulating urokinase receptor as a cause of focal segmental glomerulosclerosis. Nat Med.

[39] Nichols B (2015). Innate immunity pathways regulate the nephropathy gene Apolipoprotein L1. Kidney Int.

[40] Divers J (2014). Gene-gene interactions in APOL1-associated nephropathy. Nephrol Dial Transplant.

[41] Cooper TA (2005). Use of minigene systems to dissect alternative splicing elements. Methods.

[42] Ju Z (2015). Role of an SNP in alternative splicing of bovine NCF4 and mastitis susceptibility. PLoS One.

[43] Tang SJ (2016). Characterization of the regulation of CD46 RNA alternative splicing. J Biol Chem.

[44] Das R (2007). SR proteins function in coupling RNAP II transcription to pre-mRNA splicing. Mol Cell.

[45] Kornblihtt AR (2006). Chromatin, transcript elongation and alternative splicing. Nat Struct Mol Biol.

[46] Erkelenz S (2013). Position-dependent splicing activation and repression by SR and hnRNP proteins rely on common mechanisms. RNA.

[47] Matera AG, Wang Z (2014). A day in the life of the spliceosome. Nat Rev Mol Cell Biol.

