# Supplementary material for: Blocking the 5′ splice site of exon 4 by a morpholino oligomer triggers APOL1 protein isoform switch
Source: Sci Rep. 2018 Jun 7;8:8739. doi: 10.1038/s41598-018-27104-x (PMC5992166; doi:10.1038/s41598-018-27104-x)

Supplementary Information

Blocking the 5' splice site of exon 4 by a morpholino oligomer triggers APOL1 protein isoform switch

Amber M. Cheatham[1], Shamara E. Davis[1], Atanu Khatua[1] & Waldemar Popik[1,2]*

[1]Meharry Medical College, Center for AIDS Health Disparities Research, Department of Microbiology and Immunology, and [2]Department of Internal Medicine, 1005 D. B. Todd Blvd, Nashville, TN 37028, USA.

Supplementary Figure 1

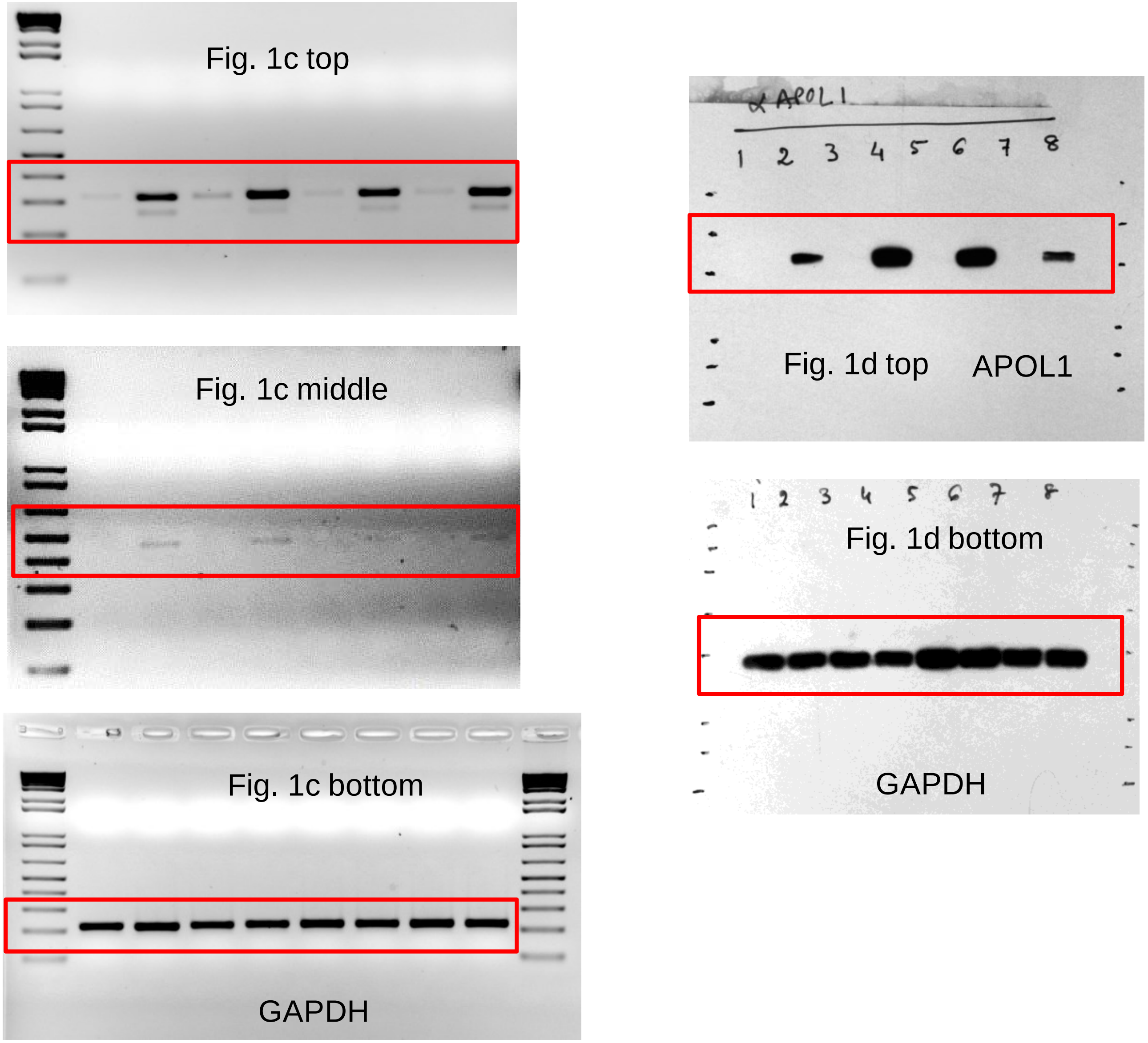

Supplementary Figure 2

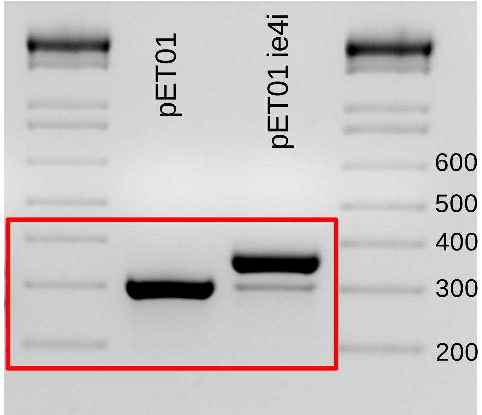

Fig. 2b top

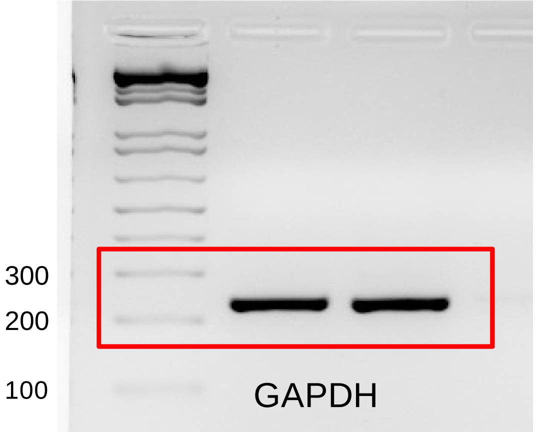

Fig. 2b bottom

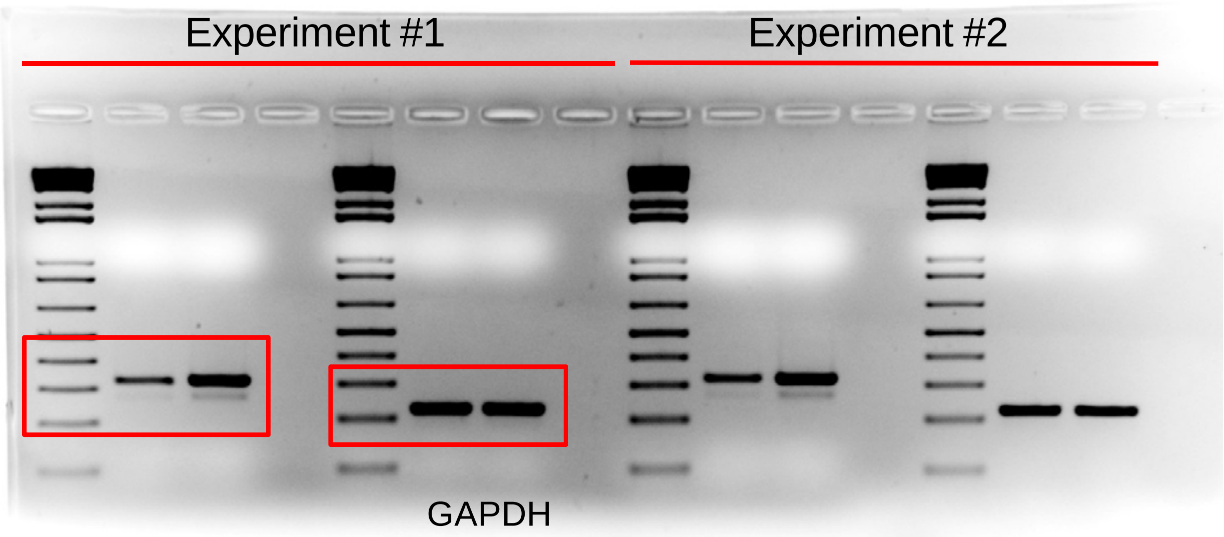

Fig. 2c top

Fig. 2c bottom

Supplementary Figure 3

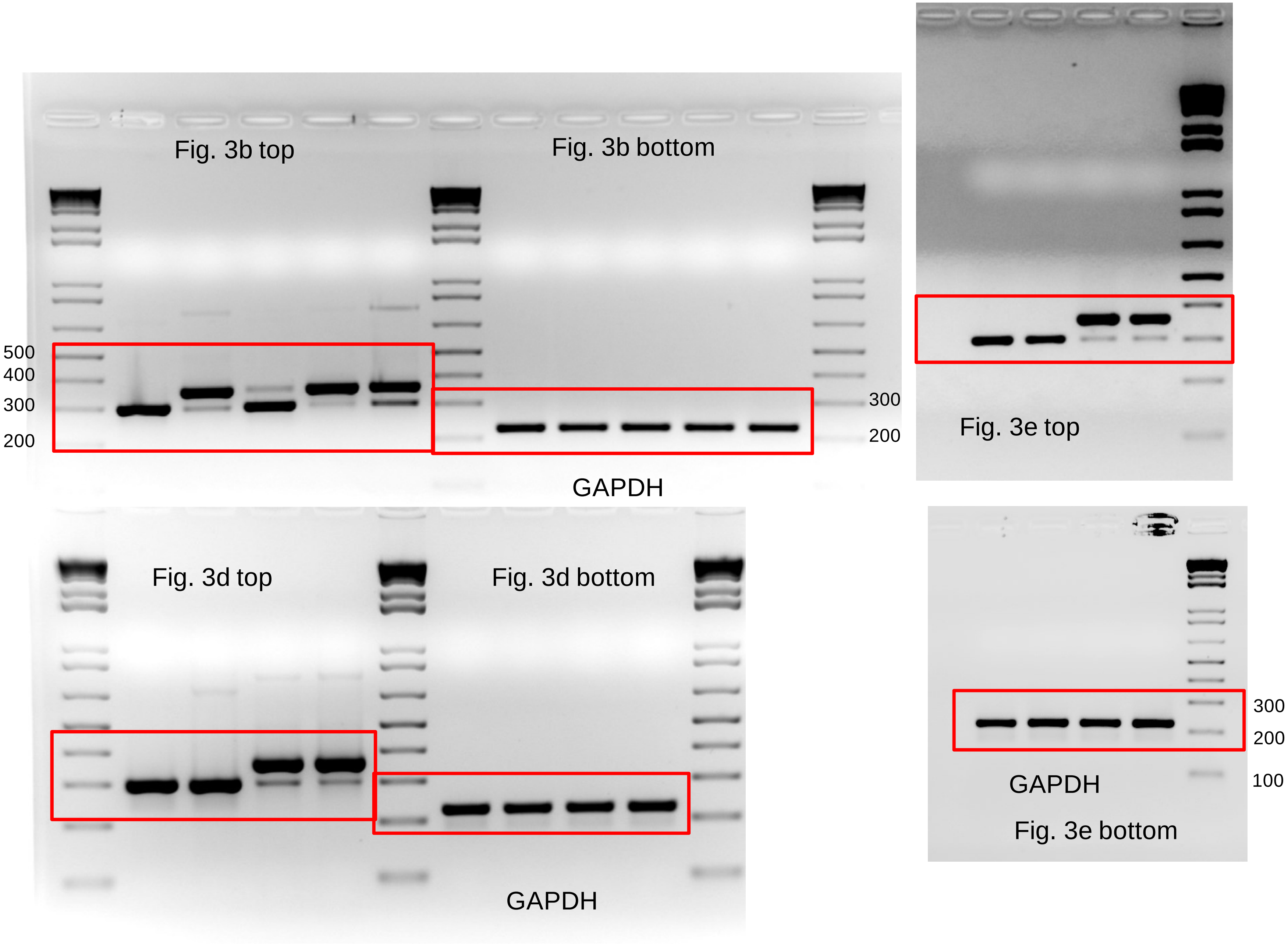

Supplementary Figure 4

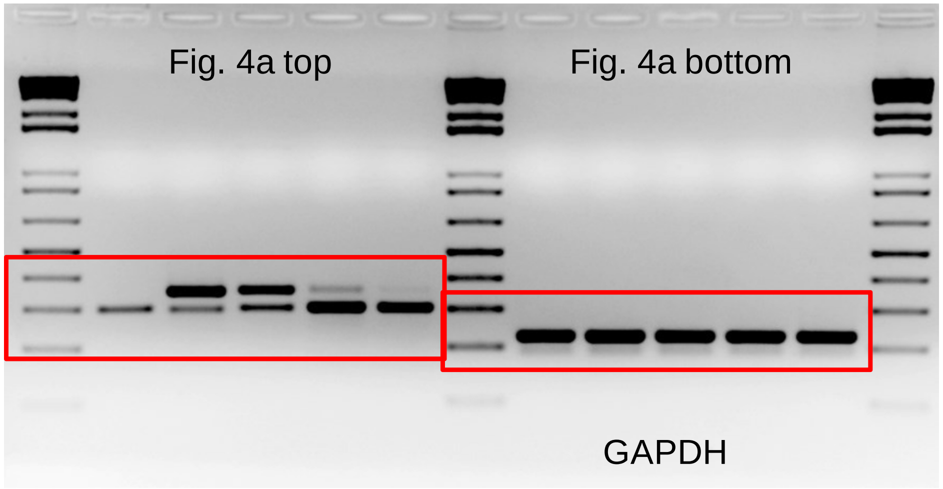

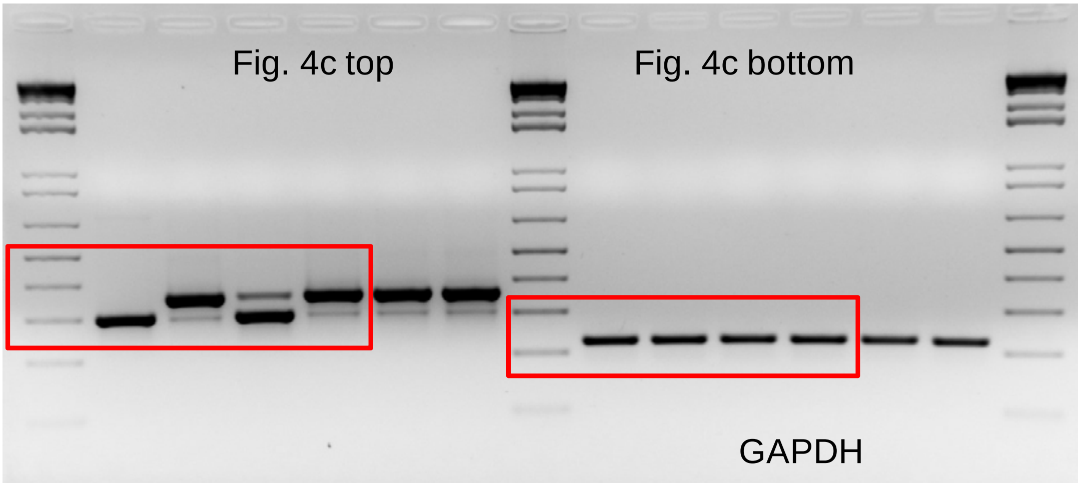

Supplementary Figure 5

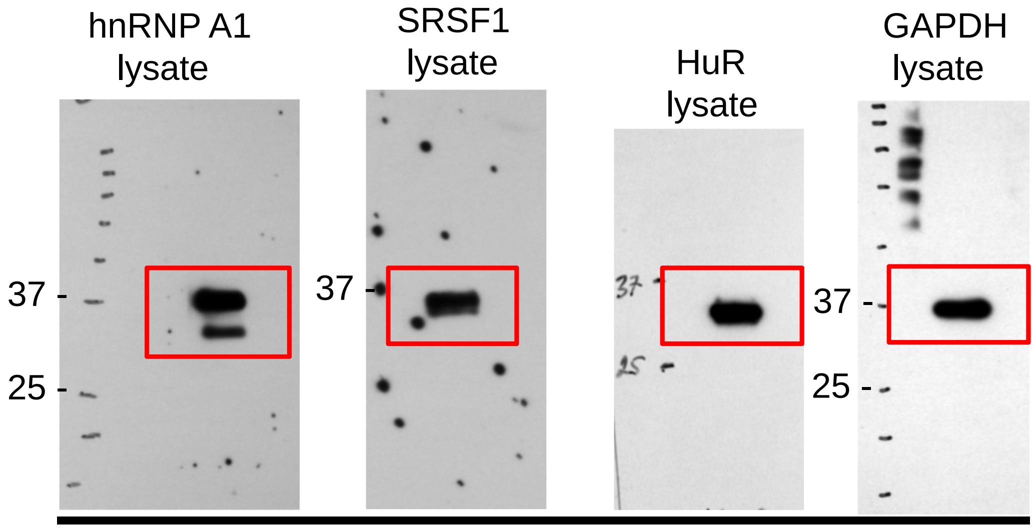

Figure 5a

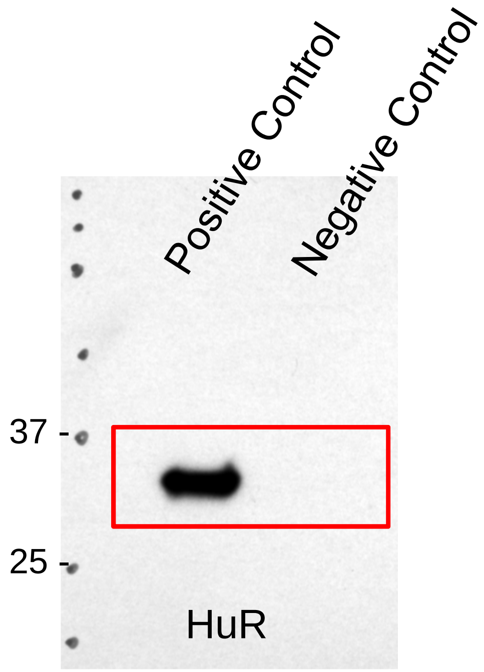

Figure 5b

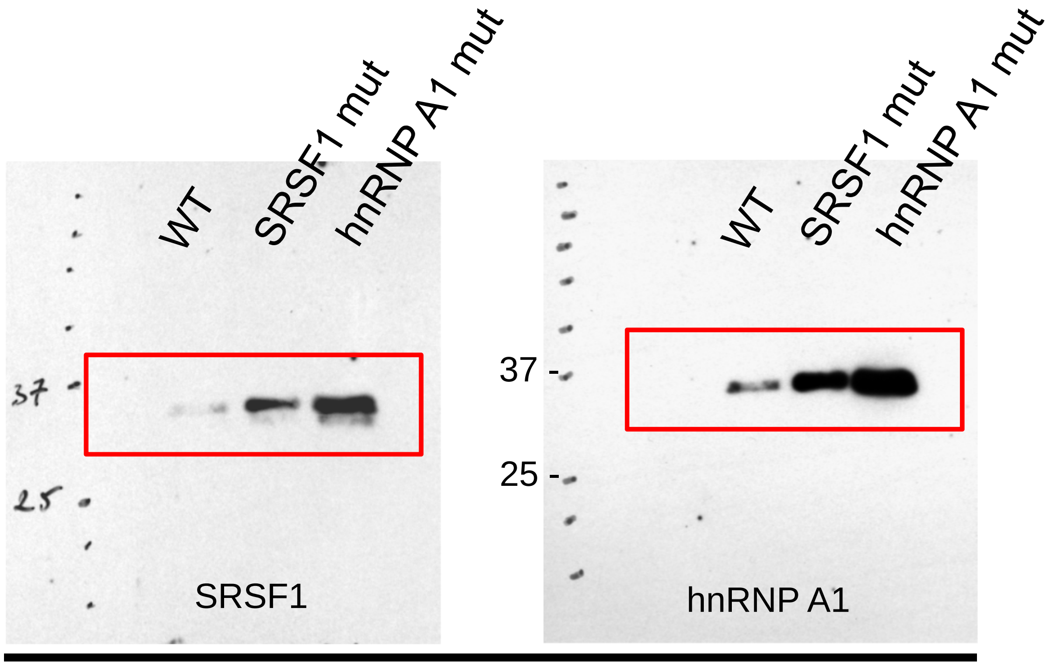

Figure 5d

Supplementary Figure 6

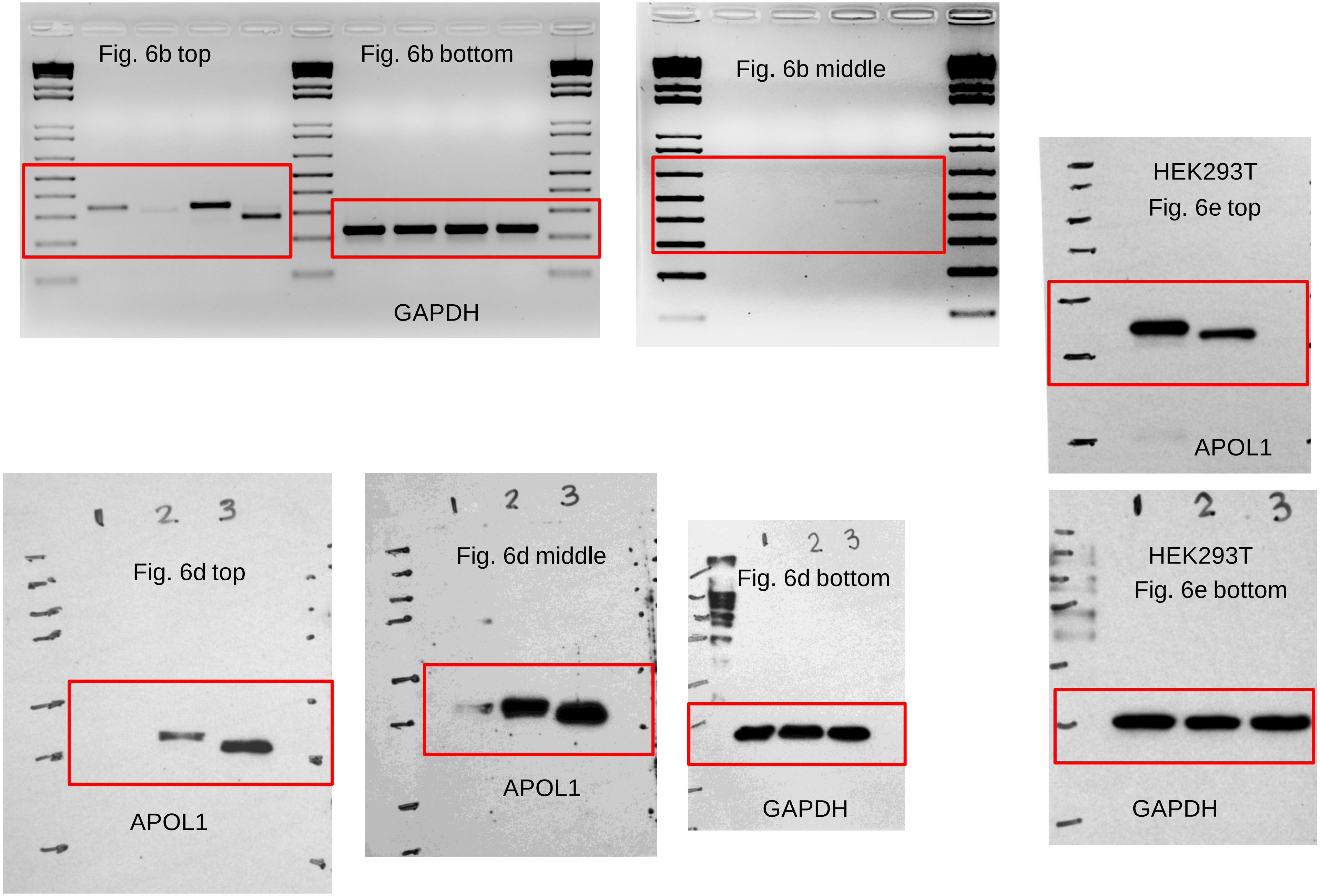

Supplement: Supplementary file 1 — Supplementary Information [file 41598_2018_27104_MOESM1_ESM.pdf]
